# “Dental Cluster” Versus “Metabolic Cluster”: Analyzing the Associations of Planned and Delivered Dental Procedures with Metabolic Syndrome, Utilizing Data from the Dental, Oral, Medical Epidemiological (DOME) Cross-Sectional Record-Based Nationwide Study

**DOI:** 10.3390/biology10070608

**Published:** 2021-06-30

**Authors:** Itzhak Abramovitz, Avraham Zini, Pablo Pribluda, Ron Kedem, Dorit Zur, Noam E. Protter, Galit Almoznino

**Affiliations:** 1 Faculty of Dental Medicine, Hebrew University of Jerusalem, Jerusalem 91120, Israel; Itzhakab@hadassah.org.il (I.A.); aviz@hadassah.org.il (A.Z.); pablopribluda@gmail.com (P.P.); 2 Hadassah Medical Center, Department of Endodontics, Jerusalem 91120, Israel; 3 Hadassah Medical Center, Department of Community Dentistry, Jerusalem 91120, Israel; 4 Medical Information Department, General Surgeon Headquarter, Medical Corps, Israel Defense Forces, Tel-Hashomer 02149, Israel; ron.kedem56@gmail.com (R.K.); Dorit48@mail.idf.il (D.Z.); 5 Chief Dental Surgeon & Head of Forensic Unit, Medical Corps, Israel Defense Forces, Tel-Hashomer 02149, Israel; noamprotter@gmail.com; 6 Hadassah Medical Center, Department of Oral Medicine, Sedation & Maxillofacial Imaging, Jerusalem 91120, Israel; 7 Big Biomedical Data Research Laboratory, Faculty of Dental Medicine, Hebrew University of Jerusalem, Jerusalem 91120, Israel

**Keywords:** metabolic syndrome, diabetes mellitus, hyperlipidemias, obesity, fatty liver, electronic health record, dental caries

## Abstract

**Simple Summary:**

There are conflicting results existing regarding the association between dental status and metabolic syndrome (MetS). This study aimed to analyze the association between the sum of the standard dental unit (SDU) scores of planned (SDU-P) and delivered (SDU-D) dental procedures per patient with MetS. Included were data from the Dental, Oral, Medical Epidemi-ological (DOME) study records-based research, which integrated large socio-demographic, medical, and dental databases of a nationally representative sample of young to middle-aged military personnel (N = 131,927). The present study demonstrated that SDU-P, but not SDU-D, is a better predictor of systemic morbidities related to MetS. In other words, MetS is associated with a higher dental treatment needs burden, rather than with dental treatments performed de facto. The study concludes that dental and general health authorities should collaborate and share in-formation and focus on reducing common health-related risk factors, such as smoking and sugar consumption, in particular among high-risk populations, such as immigrants and those with lower SES and rural locality.

**Abstract:**

There are conflicting results existing regarding the association between dental status and metabolic syndrome (MetS). The present research analyzed the associations of the sum of the standard dental unit (SDU) scores of planned (SDU-P) and delivered (SDU-D) dental procedures per patient with MetS components, consequences, and related conditions. The SDU score of each dental procedure represents the time and complexity of the executed procedure. This cross-sectional study analyzed data from the Dental, Oral, Medical Epidemiological (DOME) repository, which includes comprehensive socio-demographic, medical, and dental databases of a nationally representative sample of 132,529 military personnel. Univariate analyses revealed that SDU-P had statistically significant positive associations with all systemic morbidities related to MetS, while the SDU-D exhibited positive associations with some of the systemic morbidities and with lower ORs. SDU-P and SDU-D were associated with worse scores of auxiliary examinations used in the assessment of MetS components. SDU-P retained significant positive associations in the multivariate analysis with impaired glucose tolerance (IGT) (OR = 7.40 (1.91–28.57)), deep vein thrombosis (DVT) (OR = 5.61 (1.53–20.83)), obstructive sleep apnea (OSA) (OR = 5.05 (2.40–10.63)), and fatty liver (OR = 1.82 (1.17–2.84)). In contrast, obesity was the only systemic parameter retaining a significant association with SDU-D following multivariate analysis (OR = 1.47 (1.23–1.76)). It can be concluded that SDU-P, but not SDU-D, is a better predictor of systemic morbidities related to MetS. In other words, MetS is associated with a higher dental treatment needs burden, rather than with dental treatments performed de facto. Dental and general health authorities should collaborate and share information and focus on reducing common health-related risk factors, such as smoking and sugar consumption, in particular among high-risk populations, such as immigrants and those with lower SES and rural locality.

## 1. Introduction

Metabolic syndrome (MetS) is an accumulation of several disorders forming ‘clustering’ of metabolic abnormalities that increase the risk of an individual developing cardiovascular disease and vascular complications, such as transient ischemic attack (TIA), stroke, and deep vein thrombosis (DVT) [1]. MetS is composed of several conditions that may co-occur in an individual, including raised plasma glucose, central obesity, dyslipidemia, and hypertension [1]. There are several definitions for MetS, including definitions by the International Diabetes Federation (IDF), Group for the Study of Insulin Resistance (EGIR), World Health Organization (WHO), American Association of Clinical Endocrinologists (AACE), and the National Cholesterol Education Program (NCEP) Adult Treatment Panel III (ATP III) [1]. All definitions require auxillary examinations to assess plasma glucose, central obesity, dyslipidemia, and hypertension, but differ in the cut-offs to define pathology [1]. The prevalence of MetS in the USA is estimated at between 22 and 30%, depending on the diagnostic criteria [2]. The global prevalence of MetS is estimated at approximately 20–25% of the world adult population, and individuals with MetS are twice as likely to die from and 3 times as likely to have a myocardial infarction or stroke compared to those without MetS [3]. MetS is also associated with obesity-related disorders, such as fatty liver [4] and obstructive sleep apnea (OSA) [5].

Associations between MetS and dental morbidities have also been documented, including associations with dental caries [6,7] and periodontitis [7,8,9]. Previous studies that analyzed the associations between MetS and dental conditions employed various methods to assess dental outcomes. Some studies analyzed the associations between MetS components and dental diagnoses such as apical periodontitis [10] and periodontitis [11] as dental outcomes. We have also analyzed the associations between MetS and the diagnosis of “SOS teeth”, which are the first priority teeth for treatment due to advanced caries reaching the pulp chamber or the presence of root fragments [12]. In other studies, instead of using pathologies as dental outcomes, the dental–MetS associations were analyzed using dental/periodontal indices as outcomes, including the Decayed, Missing, and Filled teeth (DMFT) index [6,7,13,14], the Community Periodontal Index (CPI) [15,16], clinical attachment loss (CAL) [16,17], plaque index (PI), gingival index (GI), pocket depth (PD) [18], and tooth mobility [19]. Previously, we explored the associations of hypertension diagnosis with dental treatment needs and actual dental treatments [20]. Other studies have also used the number of teeth in need of dental restorations, root canal treatments, and extractions as a proxy for oral health [21]. The Atherosclerosis Risk in Communities (ARIC) study employed a self-reported history of root canal treatments as an outcome to study the associations of endodontic therapy with elevated risk of coronary heart disease, ischemic stroke, heart failure, or venous thromboembolism [22]. The wide use of indices, such as DMFT, CPI and CAL, is because some of the pathologies, such as caries, are measured as continuous parameters rather than a dichotomized diagnosis (presence of caries: yes/no). Interestingly, in the DMFT, which is the most commonly used epidemiological index for assessing dental caries, the filled and the missing teeth in this index represent outcomes of pathologies rather than the pathological process per se. Yet, associations between MetS and the incidence of tooth loss were found [23,24,25]. Economic outcomes of MetS–dental associations, such as outpatient days and costs [26] and monthly health care cost [27], were also studied.

Conflicting results exist regarding the associations between dental status and MetS. According to a recent review discussing 100 years of periodontal medicine, the majority of cross-sectional, case-controlled, or longitudinal studies revealed positive associations between poor periodontal status and cardiovascular disease and deficient diabetic control. On the other hand, the findings from randomized controlled trials testing the effects of periodontal therapy on systemic health outcomes were conflicting and inconsistent [28]. Moreover, findings from the Atherosclerosis Risk in Communities (ARIC) study did not support an independent association between endodontic treatment and the development of coronary heart disease, ischemic stroke, heart failure, or venous thromboembolism [22]. In our previous publications exploring the associations of MetS with periodontitis [11] and with caries [12,20], we have shown that the associations between dental pathologies and MetS components were only found in the univariate analyses, and they vanished following the multivariate analysis.

Furthermore, the underlying mechanisms for systemic–periodontal associations are not completely understood, although inflammatory mechanisms seem to play an important role. For example, a genome-wide association study (GWAS) for interleukin-1β levels in gingival crevicular fluid identified IL37 variants that modulate the inflammatory cascade in periodontal disease [29]. It has been reported that several pro-inflammatory cytokines, including IL-1, -6, and -8, upregulate the secretion of MMP-3, which increased with the progression of periodontitis and was involved in the regulation of IL-1β expression in gingival tissues [14].

Interestingly, some controversy exists as to whether the MetS cluster is a unique entity, since management of the syndrome is simply the treatment for each of its components [30]. However, all agree that the presence of one component of MetS is an indication to evaluate the other risk factors [30]. Regardless of whether MetS is a unique entity, individual components need to be identified and managed in order to decrease the associated morbidity and mortality [31]. Indeed, when assessing dental–systemic association studies, it is important to differentiate between studies on one systemic condition related to MetS and those including multiple co-existing systemic conditions related to MetS. Similarly, when assessing dental–systemic associations, distinguishing between studies on one dental morbidity and those considering several concomitant dental pathologies can provide valuable insights into the relationships between the entities. This need is greater because of the above-mentioned conflicting results that were found when assessing the associations between single dental pathologies (such as caries or periodontitis) with metabolic morbidities.

In light of this unmet need, the primary objective of the present study was to explore the hypothesis that a higher burden of dental treatments, rather than a single dental pathology, is positively associated with MetS components, consequences, and related conditions among a nationally representative sample of young and middle-aged adults. In this study, MetS components, consequences, and related conditions included hypertension, diabetes mellitus, impaired glucose tolerance (IGT), hyperlipidemia, cardiovascular disease, obesity, fatty liver, OSA, TIA, stroke, and DVT. We also incorporated auxiliary test results, including blood tests, used in the assessment of MetS components. Considering that one of the proposed mechanisms of association is systemic inflammatory sequelae, C-reactive protein (CRP) levels were also analyzed.

The dental burden is a function of the number of planned and delivered dental procedures and the time and complexity of the executed procedures. This concept of the accumulation of dental treatments is similar to the concept of the “MetS cluster”, which considers the simultaneous existence of multiple pathologies in an individual. While the notion of “clustering” has been examined extensively in the medical literature regarding MetS, the concept of “clustering” is rare in the context of dentistry, including analysis of the associations between a “dental cluster” and a “MetS cluster”. The literature review revealed the various methods employed to assess dental outcomes in studies assessing the associations between MetS and dental conditions. These methods include assessment of dental pathologies by using diagnoses (yes/no), as well as various dental/periodontal indices, assessment of dental treatment needs, economic outcomes, and even self-reported measures. Considering the need to assess the impact of several concomitant dental pathologies, the “dental cluster” components in the present study included assessment of dental treatment needs as well as actual dental treatments of the following: (1) fillings, (2) endodontic treatments, (3) post fabrications, (4) crowns, (5) extractions, (6) scaling and root planning, (7) and dental implants. The use of these variables as a proxy for oral health was described previously [20,21]. We hypothesized that MetS will be positively associated with higher dental treatment needs (representing presence of pathologies requiring treatments) rather than with dental treatments performed de facto.

The secondary objectives were to account for common socio-demographic and health-related risk factors for both dental and systemic morbidities by including these risk factors in the multivariate analysis.

## 2. Study Methods

### 2.1. Data Source

The current research is part of the *Dental, Oral, Medical Epidemiological (DOME)* cross-sectional record-based nationwide study [12,20,32,33]. The protocol and study methods of the DOME study have been detailed previously [33]. The DOME structured repository brings together comprehensive socio-demographic, health-related habits, dental, and medical data of a nationally representative sample of military personnel from the Israel Defense Forces (IDF). In Israel, military service is mandatory for all eligible citizens, and, therefore, the military population in Israel is large and creates a well-grounded data source for epidemiologic research among young and middle-aged adults [12,20,32,33,34]. It is noteworthy that medically complex individuals also enlist and perform non-combat tasks, and those considered unfit for physical or mental reasons can apply for voluntary tasks [12,33].

Data for the DOME repository were drawn from three military databases: (a) *DPR—Dental Patient Record*—captures the complete dental records of the patients, including all planned and delivered dental treatments; (b) *IDF’s central demographic database*—captures socio-demographic records of the military population, and (c) *CPR (Computerized patient record)*—captures general medical records [33]. Data extraction was performed by the Medical Information department of the Medical Corps, and the final database is anonymous [33].

### 2.2. Ethical Approval

The research received ethical approval from the Medical corps Institutional board (number 1281–2013) and was exempt from the requirement for informed consent because of the anonymous data analysis.

### 2.3. Study Population and Eligibility Criteria

The DOME repository utilized in this research includes military personnel meeting the following inclusion criteria: (a) attended military dental clinics of the IDF between January 2015 and January 2016, (b) aged 18–50 years, and (c) data on the individual are available from the socio-demographic, DPR, and CPR databases. Exclusion criteria were an absence of data regarding the individual (>5% missing data) from the socio-demographic, DPR, and CPR databases.

### 2.4. Definitions of Variables

#### 2.4.1. Definition of Dependent Variable: Standard Dental Unit (SDU)

In order to employ the concept of “clustering” in dentistry, a standardized scoring system that quantifies dental needs and their burden is needed. To that end, several scoring methods were developed to transform descriptions of dental diagnoses and procedures used in the electronic files into universal dental code numbers. These scoring systems have been used for statistical analysis, reimbursement, and knowledge-based and decision support. In dentistry, the American Dental Association (ADA) developed the SNO-DDS (Dental Diagnostic System) that captures patient dental diagnoses [35]. For reimbursement in dentistry, the ADA developed a procedural coding system termed Current Dental Terminology (CDT), i.e., ADA-CDT [36].

The dental database of the IDF captured in the DOME study employs standardized codes for dental procedures that are equivalent to the nomenclature of the ADA-CDT [36] as described in detail in our DOME protocol publication [33]. Similarly to the CDT, in this scoring system, each dental procedure is given a descriptive term representing the procedure (see Table 1).

Since the dental and medical treatments in the IDF are free of charge for the patients, there are no reimbursement charges for a dental procedure in the IDF. Instead, for several decades, the IDF dental corps routinely employs a scoring system for dental procedures, termed the standard dental unit (SDU). The SDU score of each dental procedure represents the time and complexity of the executed procedure. For example, the SDU score of a “dental filling” procedure (amalgam or composite) is 1, the SDU score of endodontic treatment of one root canal is 1.25, while the “crown” procedure has an SDU score of 4. The definitions and comparisons of the SDU scores and CDT-related coding are presented in Table 1.

From the database, we captured both the detailed treatment plan (SDU-P) as well as the actual treatments that were performed de facto (SDU-D). The SDU-P score sums the detailed treatment plan, i.e., the dental treatment needs, while the SDU-D sums the actual treatments performed. The sum of the SDU scores of all planned (SDU-P) and delivered (SDU-D) dental procedures per patient is used by the IDF dental corps to quantify the burden of both planned and delivered dental procedures. For example, the SDU-P “Amalgam—one surface” means the presence of a tooth in need of a filling on one surface, while for the “SDU-D”, it means the actual performance of this one surface filling. Similarly, the SDU-P “Amalgam—two surfaces”, “Amalgam—three surfaces”, and “Amalgam—four surfaces” mean the need for a filling on two, three, or four tooth surfaces, respectively, while for “SDU-D”, these definitions would account for the actual performance of the fillings. Regarding scaling: for SDU-P it means the presence of plaque and calculus that require scaling and for the SDU-D it means the actual performance of scaling to remove the plaque and calculus. The same principle accounts for the rest of the definitions: the SDU-P captures dental treatment needs, and the SDU-D captures actual dental treatments of fillings, endodontic treatments, post fabrications, crowns, extractions, scaling and root planning, and dental implants.

The sum of the SDU scores is also measured per dentist to assess the productivity of the dentists. In the present study, SDU-P and SDU-D were used as dependent variables to explore their associations with MetS components, consequences, and related conditions among a nationally representative sample of young and middle-aged adults.

#### 2.4.2. Definitions of Independent Variables

##### Socio-Demographic Variables

Information regarding the socio-demographic parameters captured in the DOME repository has been detailed in the DOME protocol paper [33]. In summary, the socio-demographic information included (1) age (years), (2) sex: male or female, (3) socio-economic status (SES): lowest (1st–4th deciles)/medium (5th–7th)/highest (8th–10th), (4) locality of residence: urban Jewish/urban non-Jewish/rural, and (f) country of birth: Western Europe, Eastern Europe, former Soviet Union (FSU), Asia, Ethiopia, Africa, North America, South America, or Israel [33,34].

##### Health-Related Habit Variables

Health-related habits were self-reported and included the following: currently smoking and teeth brushing once a day or more (yes/no); cariogenic dietary habits (e.g., snacks, sweets, etc., between/instead of meals); and sweetened beverage consumption (≥cup/day) [33,34].

##### General Health Status Variables

Assessment of the MetS cluster was based on medical diagnoses and the auxiliary test results as follows:

A. Medical diagnoses: The medical diagnoses of MetS components, consequences, and associated morbidities were retrieved from the CPR (computerized patient record) as described previously [12,20,33] and included hypertension, diabetes mellitus, impaired glucose tolerance (IGT), hyperlipidemia, cardiovascular disease, obesity, fatty liver, obstructive sleep apnea (OSA), S/P (status post) transient ischemic attack (TIA), S/P stroke, and S/P deep vein thrombosis (DVT).

B. Auxiliary test results: The auxiliary test results retrieved from the CPR included blood tests used in the assessment of MetS components as described previously [12]: weight (in kilograms), body mass index (BMI), C-reactive protein (CRP) (mg/L), glycated hemoglobin (HbA1c) (%), fasting glucose (mg/dL), cholesterol (mg/dL), high-density lipoprotein (HDL) (mg/dL), low-density lipoprotein (LDL) (mg/dL), triglycerides (mg/dL), very low density lipoprotein (VLDL) (mg/dL), and non-HDL cholesterol (mg/dL).

### 2.5. Statistical Analysis

Statistical analyses were performed using IBM^®^ SPSS^®^ software version number 26.0 (Chicago, IL, USA).

*Descriptive statistics:* The continuous variables are presented as means and standard deviations. The categorical variables are presented as absolute numbers and percentages.

*Explanatory statistics—univariate analysis*: The associations and correlations of SDU-P and SDU-D as the dependent variables with the various independent socio-demographic parameters; health-related habits; and MetS components, consequences, and associated morbidities; and auxiliary examinations were analyzed with Spearman’s correlation (for continuous variables) and non-paired t-test or ANOVA (for categorical parameters).

Linear regression models were used to calculate odds ratios (ORs) and 95% confidence intervals (CIs).

*Explanatory statistics—multivariate analysis and multicollinearity tests:*

Following the univariate analyses, multivariate linear regression analysis was performed using variables with a statistically significant result in the univariate analysis. ANCOVA was performed to assess which of the statistically significant independent variables retain their significant association, with SDU-P and SDU-D as the dependent variables. This study hypothesized that MetS components, consequences, and associated morbidities will retain statistically significant associations with SDU-P, but not with SDU-D, even after adjustment for socio-demographic parameters and health-related habits.

A *p* value <0.01 (2-tailed) was considered to indicate statistical significance in the univariate as well as multivariate analyses due to the large sample size. Multicollinearity tests were included in the multivariate linear regression model. The variance inflation factors (VIFs), which are 1/Tolerance, were calculated in the multicollinearity statistics. Although a result of VIF > 10 is regarded as indicating multicollinearity, there is a concern when VIF > 2.5, particularly in weaker models, and, for this reason, the cut-off for VIF in the current research was set at 2.5.

## 3. Results

This research included the records of 132,529 dental attendees with a mean age of 21.88 ±6.02 years. The mean SDU-P score was 2.94 ± 4.61, median 1.25, and the mean SDU-D score was 2.74 ± 3.80, median 1.25.

### 3.1. The Associations of SDU-P and SDU-D with Socio-Demographic Parameters and Health-Related Habits

Table 2 presents the associations of SDU-P and SDU-D with socio-demographic parameters and health-related habits. The *p* value determines whether the differences between the means of SDU-P and SDU-D are significant for each independent variable. The *p* value is a result of non-paired *t*-tests (for categorical independent variables with two categories), ANOVA (for categorical independent variables with more than two categories), or Spearman’s correlation (for independent continuous parameters). The odds ratio (OR) and 95% confidence interval (CI) were calculated using the general linear model to quantify the strength of the association between the SDU-P and SDU-D and the independent variables (Table 2).

#### 3.1.1. The Associations of SDU-P and SDU-D with Socio-Demographic Parameters

The SDU-P and SDU-D were positively associated with the socio-demographic parameters as follows (Table 2):

##### SDU-P and Socio-Demographic Parameters

SDU-P was positively associated with (from the highest to the lowest odds ratios (ORs)) (Table 2): rural locality (OR and 95% confidence interval (CI) = 9.53 (6.55–13.87)); birth country in Africa (OR = 5.05 (3.11–8.22)); low SES (OR = 4.99 (4.41–5.66)); birth country in Eastern Europe and FSU (OR = 4.59 (3.69–5.72)), Western Europe (OR = 2.72 (2.48–2.98)), and Asia (OR = 2.57 (1.72–3.84)); birth country Ethiopia (OR = 2.02 (1.66–2.45)); medium SES (OR = 1.97 (1.87–2.08)); age (OR = 1.29 (1.28–1.29)); and male sex (OR = 1.25 (1.18–1.33)) (Table 2).

SDU-P was negatively associated with (from the highest to the lowest ORs): urban non-Jewish locality (OR = 0.62 (0.58–0.67)) and birth country in North America (OR = 0.29 (0.24–0.34)) (Table 2).

##### SDU-D and Socio-Demographic Parameters

SDU-D was positively associated with (from the highest to the lowest ORs): low SES (OR = 12.53 (11.32–13.8)), birth country in Eastern Europe and FSU (OR = 3.98 (3.32–4.77)) and Western Europe (OR = 2.31 (2.14–2.49)), medium SES (OR = 2.16 (2.07–2.25)), birth country Ethiopia (OR = 2.09 (1.78–2.45)), and male sex (OR= 1.08 (1.03–1.13)) (Table 2).

SDU-D was negatively associated with (from the highest to the lowest ORs): birth country in South America (OR = 0.69 (0.54–0.88)), urban non-Jewish (OR = 0.56 (0.53–0.60)), and birth country in North America (OR = 0.32 (0.27–0.36)) (Table 2).

#### 3.1.2. The Associations of SDU-P and SDU-D with Health-Related Habits

SDU-P and SDU-D were positively associated with health-related habits, except for the cariogenic diet for SDU-P, as follows (from the highest to the lowest ORs and 95% CI) (Table 2): smoking (SDU-P: OR = 49.29 (44.16–55.02); SDU-D: OR = 2.96 (2.70–3.25)), brushing teeth less than once a day (SDU-P: OR = 3.84 (3.51–4.19); SDU-D: OR = 2.06 (1.92–2.20)), consumption of sweetened beverages (SDU-P: OR = 1.17 (1.09–1.27); SDU-D: OR = 2.14 (2.00–2.30)), and consumption of cariogenic diet (SDU-P: non-significant; SDU-D: OR = 1.58 (1.48–1.70)) (Table 2).

### 3.2. Associations of SDU-P and SDU-D with Metabolic Syndrome (MetS) Components, Consequences, and Related Conditions

Table 3 presents analyses of the associations of SDU-P and SDU-D with MetS components, consequences, and related conditions. SDU-P and SDU-D were positively associated with systemic morbidities related to MetS, except for the associations of the SDU-D with OSA, stroke, and DVT. In all analyzed morbidities, the SDU-P exhibited higher ORs compared to the SDU-D as follows (from the highest to the lowest ORs and 95% CI) (Table 3): IGT (SDU-P: OR = 124.84 (56.12–277.72); SDU-D: OR = 2.47 (1.28–4.77)), TIA (SDU-P: OR = 116.03 (46.74–288.03); SDU-D: OR = 5.06 (2.39–10.70)), OSA (SDU-P: OR = 76.08 (45.81–126.37); SDU-D: non-significant), diabetes mellitus (SDU-P: OR = 60.96 (37.45–99.23); SDU-D: OR = 2.58 (1.72–3.85)), fatty liver (SDU-P: OR = 60.94 (45.34–81.89); SDU-D: OR = 1.46 (1.14–1.86)), obesity (SDU-P: OR = 41.57 (37.39–46.22); SDU-D: OR = 2.32 (2.13–2.54)), DVT (SDU-P: OR = 58.99 (24.70–140.89); SDU-D: non-significant), hyperlipidemia (SDU-P: OR = 24.46 (18.26–32–77); SDU-D: OR = 2.03 (1.59–2.58)), stroke (SDU-P: OR = 23.54 (9.16–60.48); SDU-D: non-significant), cardiovascular disease (SDU-P: OR = 9.02 (7.74–10.50); SDU-D: 1.28 (1.13–1.46)), and hypertension (SDU-P: OR = 8.11 (6.93–9.49); SDU-D: 1.69 (1.49–1.93)) (Table 3).

#### The Correlations of SDU-P and SDU-D with Auxiliary Test results Including Blood Tests Used in the Assessment of MetS Components

Table 4 presents the correlations of SDU-P and SDU-D with auxiliary examinations. SDU-P and SDU-D exhibited weak positive correlations with all auxiliary examinations, except for HDL, which had negative correlations with SDUs. In all auxiliary examinations analyzed, SDU-P exhibited higher ORs than SDU-D, although the correlations had ORs close to 1 and weak Spearman’s rho coefficients. The highest ORs were noted for the correlations with glycated hemoglobin (HbA1c) (SDU-P: OR = 2.558 (1.733–3.775); SDU-D: OR = 1.324 (1.106–1.585)).

### 3.3. Multivariate Analyses

Following the univariate analyses, multivariate linear regression analyses were performed for SDU-D (Table 5) and SDU-P (Table 6) and as dependent variables with statistically significant independent parameters that were entered simultaneously into the analysis. Collinearity statistics were performed and the results ruled out collinearity (VIF < 2.5) (see Table 5 and Table 6). Due to multicollinearity, blood examinations were not entered simultaneously with metabolic morbidities into the multivariate analysis.

#### 3.3.1. Multivariate for SDU-P as the Dependent Variable

The following independent parameters retained a statistically significant association with SDU-P following multivariate analysis (from the highest to the lowest ORs) (Table 6): IGT (OR = 7.40 (1.91–28.57)), DVT (OR = 5.61 (1.53–20.83)), OSA (OR = 5.05 (2.40–10.63)), low vs. high SES (OR = 3.76 (3.12–4.54)), birth country: Eastern Europe and FSU vs. native Israeli (OR = 3.43 (2.52–4.67)), birth country: Western Europe vs. native Israeli (OR = 2.26 (1.97–2.58)), birth country: Ethiopia vs. native Israeli (OR = 1.93 (1.45–2.57)), medium vs. high SES (OR = 1.85 (1.71–1.99)), fatty liver (OR = 1.82 (1.17–2.84)), brushing teeth less than once a day (OR = 1.58 (1.42–1.77)), smoking (OR = 1.36 (1.12–1.64)), consumption of sweetened beverages (OR = 1.31 (1.22–1.41)), and age (OR = 1.19 (1.18–1.20)) (Table 4).

The locality of residence of urban non-Jewish vs. urban Jewish was negatively associated with SDU-P (OR = 0.70 (0.63–0.78)) as well as birth country: North America vs. native Israeli (OR = 0.52 (0.40–0.68)) (Table 4).

#### 3.3.2. Multivariate for SDU-D as the Dependent Variable

The following independent parameters retained a statistically significant association with SDU-D following multivariate analysis (from the highest to the lowest ORs) (Table 5): low vs. high SES (OR = 9.84 (8.23–11.77)), birth country: Eastern Europe and FSU vs. native Israeli (OR = 4.45 (3.32–5.96)), birth country: Western Europe vs. native Israeli (OR = 2.19 (1.93–2.48)), brushing teeth less than once a day (OR = 2.11 (1.90–2.35)), birth country: Ethiopia vs. native Israeli (OR = 2.03 (1.54–2.65)), medium vs. high SES (OR = 1.95 (1.81–2.09)), consumption of sweetened beverages (OR = 1.89 (1.75–2.05)), obesity (OR = 1.47 (1.23–1.76)), smoking (OR = 1.25 (1.04–1.50)), consumption of cariogenic diet (OR = 1.13 (1.05–1.23)), and age (OR = 1.012 (1.004–1.020)) (Table 5).

SDU-D was negatively associated with locality of residence: urban non-Jewish vs. urban Jewish (OR = 0.61 (0.55–0.67)) and birth country: North America vs. native Israeli (OR = 0.45 (0.35–0.58)) (Table 5).

## 4. Discussion

The present research analyzed the associations of SDU-P (planned) and SDU-D (delivered) scores per subject with MetS components, consequences, and related conditions in a nationally representative sample of young and middle-aged adults. The study demonstrated that the total SDU-P, but not SDU-D, is associated with MetS. The SDU score takes into account the time and complexity of the procedure and represents the procedural burden. Univariate analyses were performed to analyze the associations between SDU-P and SDU-D and MetS components, consequences, and related conditions. The univariate analyses revealed that the SDU-P had statistically significant positive associations with all the systemic morbidities related to MetS that were analyzed. On the other hand, the SDU-D only exhibited positive associations with some of the systemic morbidities in the univariate analyses, and with lower ORs compared to the SDU-P.

To adjust for important confounding parameters, following the univariate analyses, we performed multivariate analyses (Table 5 and Table 6). The multivariate analysis of SDU-P confirmed the study hypothesis, since the SDU-P retained its statistically significant associations with the following systemic morbidities related to MetS: IGT, DVT, OSA, and fatty liver (Table 6). In contrast, obesity was the only systemic parameter that retained a significant association with the SDU-D following multivariate analysis (Table 5).

Overall, the results confirmed the study hypothesis that the SDU-P and not the SDU-D is associated with systemic morbidities related to MetS. In other words, MetS is associated with a higher dental treatment needs burden rather than with the burden of the dental treatments performed de facto. While the SDU-P represents an accumulation of dental problems of an individual, the SDU-D represents completed treatments. While the SDU-P represents existing pathologies requiring treatments, the SDU-D counts completed treatments that depend on external influencing factors, such as patient compliance, availability of treatment, treatment experience, and specialty of the treating dental professional. This means that the more teeth that require treatments and the more complex the required treatments are in terms of time and complexity, the higher the likelihood of metabolic diseases. The present study is one of few studies that analyzed the associations between dental burden and MetS. For example, a recent study found that combinations of BMI levels and metabolic dysfunctions were significantly associated with dental outpatient days and costs [26]. Vice versa, monthly health care cost for MetS per capita decreased in municipalities in Japan that could afford to implement dental health programs [27].

**Associations of SDU-P and SDU-D with socio-demographic parameters.** Common risk factors of dental morbidities shared with MetS include, among others, age, race, low household income, weight, abdominal obesity, smoking, and a carbohydrate-rich diet [37,38]. For this reason, it is extremely important to adjust for these common risk factors when analyzing the associations between dental morbidities and MetS. The SDU-P and SDU-D were both positively associated with well-known socio-demographic and health-related risk factors, establishing a patient profile with higher SDU scores that includes the following: older age; male sex; lower SES; rural locality; immigration from Western Europe, Eastern Europe, FSU, and Ethiopia; brushing teeth less than once a day; and consumption of sweetened beverages.

Age had a positive association with SDUs, an expected finding considering that dental morbidities are time dependent and coincide with augmented morbidity over time as suggested by the findings of Gordon et al. [37] and Kruger et al. [39]. Men exhibited higher SDU scores in this study. Previous studies demonstrated conflicting results: some demonstrated higher prevalence in men similar to our findings [40], while others concluded that women have a higher caries risk than men [41]. The latter finding was attributed to societal and cultural changes, which have narrowed over the past 20 years [42]. A higher SDU among men could be attributed to dental trauma among men in general and particularly among the military population [32]. The associations between lower SES [43] and rural locality [44] with higher dental needs have been described previously. The current study also adjusted for country of birth, since immigration is an important issue in epidemiology considering the “healthy immigrant effect” [45]. This effect refers to the finding that immigrants are in relatively better health on arrival in Canada/US compared to native-born individuals and that immigrant health converges with native-born levels over time [45]. Israel is an immigrant state, with the largest increase in immigrants in the 1990s from the FSU and Ethiopia. Indeed, the current study demonstrated that some immigrant populations exhibited higher SDU scores compared to native Israelis, including Western Europe, Eastern Europe, FSU, and Ethiopia.

**Associations of SDU-P and SDU-D with health-related habits.** As expected, higher SDU scores were found among those who brush their teeth less than once a day and those with a higher consumption of cariogenic compounds, in particular sweetened beverages. These findings are from well-known and established research that demonstrated the contribution of poor oral hygiene [46], consumption of a cariogenic diet [47], and sweetened beverages [48] to the development of dental pathology.

Smoking was positively associated with both SDUs. Smoking has long been established as an oral health modifier and related to periodontitis [49], caries [50], tooth loss [51], and oral cancer [52]. In line with our findings, more severe periodontitis cases were noted in smokers with a family history of myocardial infarction [53]. Smoking is a common risk factor for both metabolic and dental/oral diseases according to the common risk factors approach [54].

**Associations of SDU scores with metabolic morbidity.** The current study findings support the hypothesis that in a manner similar to the concept of the MetS cluster, higher SDU-P scores (representing the accumulation of dental pathologies) are associated with some of the MetS components, consequences, and related conditions independent of the socio-demographic and health-related risk factors analyzed. Auxiliary examinations were performed on several thousand patients, and metabolic morbidity was diagnosed in several hundred of them. The auxiliary examination results included normal as well as pathological values, and worse scores were positively associated with accumulations of dental pathologies.

**Associations of SDU scores with TIA, stroke, and DVT.** The findings suggest that higher SDU-P scores were associated with more vascular events, such as venous thrombosis (DVT) and arterial thrombosis/hemorrhage (TIA, stroke). In line with our findings, others found associations of stroke with periodontitis [55] and tooth loss [56] and between periodontitis and recurrent vascular events in stroke/TIA patients [57]. Moreover, bacterial DNA from viridans streptococci, mostly of oral origin, was found in the aspired thrombi of patients with acute ischemic stroke [58]. These associations have been attributed to systemic inflammation and platelet activation that speed up the development of atherothrombotic lesions [58].

**Associations of SDU scores with diabetes mellitus and impaired glucose tolerance (IGT).** In the present study, the SDU-P was particularly associated with IGT, a pre-diabetic stage. A prediabetic condition is characterized by an impaired glucose tolerance test (IGT), impaired fasting blood glucose (IFG), and higher than normal hemoglobin A1c (HbA1c) [59]. In congruence with our results, other studies reported that prolonged poor glycemic control has been associated with a higher risk of caries [60] and periodontal disease progression [61]. Moreover, periodontal infection was positively associated with prevalent IGT in a nationally representative sample in the USA [62].

**Associations of SDU with obstructive sleep apnea (OSA).** OSA retained a statistically significant association with SDU-P. The syndrome is one of the most serious sleep disorders in terms of morbidity and mortality [63]. Nightly use of continuous positive airway pressure (CPAP) to physically maintain an open upper airway is the standard first-line therapy, but long-term use of CPAP can have negative consequences for oral health, such as dry mouth and incisor retroclination [64]. An alternative is a mandibular advancement device, but in order to avoid dental damage from the device, poor oral health, such as dental caries, abscesses, or periodontal diseases, should be managed before using mandibular advancement therapy [64].

**Associations of SDU with obesity.** Obesity was positively associated with SDU-P and SDU-D. Obesity is one of the leading preventable causes of death worldwide, with increasing rates in adults and children [65]. The present study highlights the importance of controlling for risk factors common to both dental and metabolic morbidities, considering the “Diabesity” pandemic and the impact of a cariogenic diet and sweetened beverage intake on obesity, diabetes, and dental diseases. Ebersole et al. [66] provided evidence connecting obesity and oral health to salivary and serum analyte levels occurring in association with cardiac events. They demonstrated that adiponectin, an anti-inflammatory molecule produced by adipose cells, was significantly lower in the serum of acute myocardial infarction patients, and salivary adiponectin decreased with increasing BMI [66]. Oral health was significantly worse in the acute myocardial infarction patients, and both serum and salivary adiponectin were higher in control subjects with better oral health. Serum CRP levels were elevated in the patients regardless of oral health [66].

**Associations of SDU with Fatty liver.** Fatty liver disease affects 20–30% of the world’s population [67] and involves the accumulation of fat in the liver [68] and pathologic consequences, such as coronary artery disease [69], hepatic cell damage, cirrhosis, or cancer [68]. The association between the triad of diabetes, fatty liver, and obesity was highlighted in a recent study showing that weight gain and re-emergence of diabetes were associated with an increase in liver-derived plasma triglyceride, re-accumulation of fat within the pancreas, and recurrence of beta-cell dysfunction [70]. In this study, we also demonstrated an association of the accumulation of dental pathologies with the triad of diabetes, fatty liver, and obesity.

**Strengths and limitations.** The main strengths of this study are its large sample size (132,529) and the strict protocol and uniform codes used. The data were based on records and therefore not impacted by patient recall bias, except for health-related habits. The comprehensive DOME repository, which combines socio-demographic, dental, and medical databases, provided a unique opportunity to examine the “dental cluster” with the “metabolic cluster” and to explore their associations while controlling for socio-demographic variables and health-related habits that were not previously considered simultaneously in this context in the medical and dental literature. Furthermore, the large sample size enabled us to capture consequences of MetS, such as TIA, stroke, and DVT, which are relatively uncommon, in particular among young to middle-aged adults.

Limitations include the cross-sectional design, which can only suggest associations and correlations but not causal relations. Although this study analyzed important covariates, considering the depth of the topic, other parameters were not analyzed, e.g., family medical history, childhood, in utero exposures, genetic parameters, and history of health-related habits. Although various ethnicities in a nationwide sample were included in the study, the sample was taken from military personnel in Israel, which may limit the generalizability of the results. There is a need for future multicenter international longitudinal research that includes genetic and epidemiological data in additional settings and populations in order to discover the origin and pathways underlying the observed associations found in the present study.

## 5. Conclusions

In conclusion, the SDU-P and not the SDU-D can be considered a predictor of systemic morbidities related to MetS. In other words, the results suggest that MetS is associated with a higher dental treatment needs burden rather than with the burden of the dental treatments performed de facto. Dental and general health authorities should collaborate by sharing the information regarding dental and systemic morbidities. The findings also highlight the importance of adopting a holistic risk management approach that focuses on reducing common health-related risk factors, such as smoking and sugar consumption, in particular among high-risk populations, such as immigrants and those with lower SES and rural locality.

## Figures and Tables

**Table 1:** Standard dental unit (SDU) scoring system: Nomenclature of the standardized dental patient record (DPR) codes for dental procedures and their equivalent nomenclature in the American Dental Association (ADA) Current Dental Terminology (CDT)-2018.

DPR Code—Abbreviations of the Procedure	Equivalent ADA CDT-2018 Code	Nomenclature	SDU Value
Dental examination	D0150	Comprehensive oral evaluation	0.25
Scaling	D4910	Periodontal maintenance	0.75
Scaling-hygienist	D4355	Full mouth debridement to enable diagnosis	0.75
Root planning	D4341	Periodontal scaling and root planning—≤4 teeth per quadrant	1.00
Periodontal examination	D0180	Comprehensive periodontal evaluation	1.00
Periodontal Treatment plan	D4999	Unspecified periodontal procedure, by report	0.5
Occlusal guard	D9940	Occlusal guard	2.0
Occlusal examination	D9950	Occlusion analysis-mounted case	0.5
Amalgam—one surface	D2140	Amalgam—one surface	1.00
Amalgam—two surfaces	D2150	Amalgam—two surfaces	1.00
Amalgam—three surfaces	D2160	Amalgam—three surfaces	1.00
Amalgam crown	D2161	Amalgam—four or more surfaces	1.5
Composite restoration	D2330, D2331 D2332, D2335	Resin-based composite—one to four surfaces	1.00
Crown restoration	D2390	Resin-based composite crown	1.5
Root canal treatment—one canal (RCT1)	D3310	Endodontic therapy, anterior tooth	1.25
RCT 2 canals	D3320	Endodontic therapy, bicuspid tooth	2.5
RCT 3 canals	D3330	Endodontic therapy, molar	3.75
RCT 4 canals	D3330	Endodontic therapy, molar	5.00
RCT 5 canals	D3330	Endodontic therapy, molar	6.25
Endodontic therapy, atypical root canal anatomy, one root canal	D3310	Endodontic therapy, anterior tooth	1.5
Endodontic therapy, atypical, 2RCT	D3320	Endodontic therapy, bicuspid tooth	3.00
Endodontic therapy, atypical, 3RCT	D3330	Endodontic therapy, molar	4.5
Endodontic therapy, atypical, 4RCT	D3330	Endodontic therapy, molar	6.00
Root canal retreatment (RTR) 1 canal	D3346	Retreatment anterior	1.5
RTR 2 canals	D3347	Retreatment bicuspid	3.00
RTR 3 canals	D3348	Retreatment molar	4.5
RTR 4 canals	D3348	Retreatment molar	6.00
RTR 5 canals	D3348	Retreatment molar	7.5
Extraction erupted tooth/ exposed root	D7140	Extraction, erupted tooth/exposed root (elevation and/or forceps removal)	1.00
Surgical extraction	D7210	Extraction erupted tooth requiring removal of bone and/or sectioning of tooth, and/or elevation of mucoperiosteal flap	1.5
Dental implant	D6010	Surgical placement of implant body	2.00
Crown	D2740, D2750 D2751, D2791 D2790, D2792	Crown—porcelain/ceramic, Crown—porcelain fused to high noble metal Crown—porcelain fused to a predominantly base metal Crown—full cast predominantly base metal, Crown—full cast noble metal	4.00
Implant-supported crown	D6065 D6066 D6067	Implant-supported porcelain/ceramic crown Implant-supported porcelain fused to metal crown Implant-supported metal crown	4.00
Implant-supported abutment	D6057	Implant-supported custom fabricated abutment	1.00
Direct post and core	D2950	Core buildup, including any pins when required	1.5
Indirect post and core	D2952	Post and core, indirectly fabricated	2.00

**Table 2:** The associations of SDU-P (planned procedures) and SDU-D (delivered procedures) with socio-demographic and health-related risk factors. # Spearman’s correlation, * non-paired *t*-test, ˄ ANOVA, ˅ general linear model, FSU: former Soviet Union.

Parameter	Variable	N	SDU-P (Planned Procedures)			SDU-D (Delivered Procedures)		
			Mean ± SD	*p* Value	OR and 95% CI ˅	Mean ± SD	*p* Value	OR and 95% CI ˅
Sex	Female	33,063	2.7 ± 4.0	<0.001 *	1	2.7 ± 3.7	0.002 *	1
	Male	99,466	3.0 ± 4.8		1.25 (1.18–1.33)	2.8 ±3.8		1.08 (1.03–1.13)
Socio-economic status (SES)	Low	5719	4.1 ± 5.7	<0.001 ˄	4.99 (4.41–5.66)	4.7 ±5.6	<0.001 ˄	12.53 (11.32–13.8)
	Medium	68,619	3.2 ± 4.8		1.97 (1.87–2.08)	3.0 ± 4.0		2.16 (2.07–2.25)
	High	56,707	2.5 ± 4.1		1	2.2 ± 3.1		1
Locality of residence	Urban Jewish	113,468	3.0 ± 4.6	<0.001 ˄	1	2.8 ± 3.8	<0.001 ˄	1
	Urban non-Jewish	17,918	2.5 ± 4.3		0.62 (0.58–0.67)	2.2 ± 3.3		0.56 (0.53–0.60)
	Rural	583	5.2 ±6.6		9.53 (6.55–13.87)	2.7 ± 3.8		0.91 (0.66–1.23)
Country of birth	Western Europe	10,571	3.9 ± 5.3	<0.001 ˄	2.72 (2.48–2.98)	3.5 ± 4.4	<0.001 ˄	2.31 (2.14–2.49)
	Eastern Europe and FSU	1715	4.4 ± 5.6		4.59 (3.69–5.72)	4.0 ± 4.9		3.98 (3.32–4.77)
	Asia	509	3.8 ± 5.4		2.57 (1.72–3.84)	2.8 ± 3.8		1.13 (0.81–1.57)
	Ethiopia	2185	3.5 ± 4.9		2.02 (1.66–2.45)	3.4 ± 4.2		2.09 (1.78–2.45)
	Africa	345	4.4 ± 6.9		5.05 (3.11–8.22)	2.6 ± 3.4		0.94 (0.63–1.41)
	North America	2859	1.6 ± 2.7		0.29 (0.24–0.34)	1.5 ± 2.3		0.32 (0.27–0.36)
	South America	957	2.6 ± 4.0		0.79 (0.59–1.06)	2.3 ± 3.5		0.69 (0.54–0.88)
	Israel	113,359	2.8 ± 4.5		1	2.6 ± 3.7		1
Brushing teeth once a day or more	No	17,820	4.2 ±6.5	<0.001 ˄	3.84 (3.51–4.19)	3.4 ±4.4	<0.001 ˄	2.06 (1.92–2.20)
	Yes	39,676	2.9 ± 4.0		1	2.7 ± 3.6		1
Cariogenic diet consumption	No	22,003	2.9 ± 4.0	0.692	1	2.6 ± 3.4	<0.001 ˄	1
	Yes	22,975	2.9 ± 4.1		1.01 (0.94–1.09)	3.1 ± 4.1		1.58 (1.48–1.70)
Sweetened beverage consumption	No	20,432	2.8 ± 4.0	<0.001 ˄	1	2.4 ± 3.2	<0.001 ˄	1
	Yes	24,487	3.0 ± 4.1		1.17 (1.09–1.27)	3.2 ± 4.1		2.14 (2.00–2.30)
Smoking	No	125,645	2.7 ± 4.2	<0.001 ˄	1	2.7 ± 3.7	<0.001 ˄	1
	Yes	6884	6.6 ± 8.4		49.29 (44.16–55.02)	3.8 ± 4.8		2.96 (2.70–3.25)
Parameter		N	Spearman’s rho coefficient #	*p* value	OR and 95% CI ˅	Spearman’s rho coefficient #	*p* value	OR and 95% CI ˅
Age		132,529	0.401	<0.001	1.29 (1.28–1.29)	0.162	<0.001	1.04 (1.03–1.04)

**Table 3:** The associations of SDU-P (planned procedures) and SDU-D (delivered procedures) with MetS components, consequences, and related conditions.

Parameter	Variable		SDU-P (Planned Procedures)			SDU-D (Delivered Procedures)		
		N	Mean ± SD	*p* Value	OR and 95% CI ˅	Mean ± SD	*p* Value	OR and 95% CI ˅
Hypertension	No	1w29,166	2.9 ± 4.5	<0.001 ˄	1	2.7 ± 3.7	<0.001 ˄	1
	Yes	3363	4.9 ± 7.2		8.11 (6.93–9.49)	3.2 ± 4.2		1.69 (1.49–1.93)
Diabetes mellitus	No	132,184	2.9 ± 4.6	<0.001 ˄	1	2.7 ± 3.8	<0.001 ˄	1
	Yes	345	7.0 ±9.1		60.96 (37.45–99.23)	3.7 ± 4.7		2.58 (1.72–3.85)
Impaired glucose tolerance (IGT)	No	132,401	2.9 ± 4.6	<0.001 ˄	1	2.7 ± 3.7	0.007 ˄	1
	Yes	128	7.7 ±9.1		124.84 (56.12–277.72)	3.6 ± 4.6		2.47 (1.28–4.77)
Hyperlipidemia	No	131,568	2.9 ± 4.5	<0.001 ˄	1	2.7 ± 3.7	<0.001 ˄	1
	Yes	961	6.1 ±7.9		24.46 (18.26–32−77)	3.4 ±4.4		2.03 (1.59–2.58)
Obesity	No	125,081	2.7 ± 4.2	<0.001 ˄	1	2.7 ± 3.7	<0.001 ˄	1
	Yes	7448	6.4 ±8.4		41.57 (37.39–46.22)	3.5 ± 4.4		2.32 (2.13–2.54)
Cardiovascular disease	No	128,931	2.9 ± 4.5	0.001 ˄	1	2.7 ± 3.8	0.001 ˄	1
	Yes	3598	5.1 ±6.9		9.02 (7.74–10.50)	2.9 ± 3.9		1.28 (1.13–1.46)
Fatty liver	No	131,591	2.9 ± 4.5	<0.001 ˄	1	2.7 ± 3.8	0.002 ˄	1
	Yes	938	7.0 ±8.5		60.94 (45.34–81.89)	3.1 ± 3.7		1.46 (1.14–1.86)
Obstructive sleep apnea (OSA)	No	132,211	2.9 ± 4.6	<0.001 ˄	1	2. 7 ± 3.8	0.533 ˄	1
	Yes	318	7.3 ± 9.3		76.08 (45.81–126.37)	2.6 ± 3.2		0.87 (0.57–1.33)
Transient ischemic attack (TIA)	No	132,430	2.9 ± 4.6	<0.001 ˄	1	2.7 ± 3.7	<0.001 ˄	1
	Yes	99	7.7 ± 7.8		116.03 (46.74–288.03)	4.4 ± 5.8		5.06 (2.39–10.70)
Stroke	No	132,437	2.9 ± 4.6	0.001 ˄	1	2.7 ± 3.8	0.059 ˄	1
	Yes	92	6.1 ± 6.1		23.54 (9.16–60.48)	3.5 ± 4.0		2.11 (0.97–4.60)
Deep vein thrombosis (DVT)	No	128,931	2.9 ± 4.6	0.001 ˄	1	2.7 ± 3.8	0.068	1
	Yes	108	7.0 ± 10.5		58.99 (24.70–140.89)	3.4 ± 4.5		1.95 (0.95–3.99)

˄ ANOVA, ˅ general linear model.

**Table 4:** The correlations of SDU-P (planned procedures) and SDU-D (delivered procedures) with auxiliary test results, including blood tests, used in the assessment of MetS components. # Spearman’s correlation, ˅ general linear model.

		SDU-P (Planned Procedures)				SDU-D (Delivered Procedures)		
Parameter	N	Mean ± SD	Spearman’s rho Coefficient #	*p* Value	OR and 95% CI ˅	Spearman’s rho Coefficient #	*p* Value	OR and 95% CI ˅
Weight (kilograms)	66,617	72.2 ± 31.7	0.105	<0.001	1.008 (1.007–1.010)	0.05	<0.001	1.002 (1.001–1.003)
Body mass index (BMI)	66,617	24.1 ± 4.5	0.129	<0.001	1.167 (1.157–1.178)	0.061	<0.001	1.057 (1.050–1.065)
C-reactive protein (CRP) (mg/L)	43,081	3.8 ± 10.1	0.085	<0.001	1.008 (1.002–1.014)	0.051	<0.001	1.005 (1.001–1.008)
Glycated hemoglobin (HbA1c) (%)	2832	5.4 ± 0.9	0.168	<0.001	2.558 (1.733–3.775)	0.048	0.033	1.324 (1.106–1.585)
Fasting glucose (mg/dL)	2927	87.1 ± 11.9	0.059	0.030	1.047 (1.016–1.080)	0.052	0.008	1.020 (1.005–1.035)
Cholesterol (mg/dL)	39,472	175.7 ± 35.9	0.096	<0.001	1.019 (1.016–1.021)	0.030	<0.001	1.003 (1.002–1.005)
High-density lipoprotein (HDL) (mg/dL)	39,453	48.5 ± 11.9	−0.084	<0.001	0.955 (0.949–0.962)	−0.087	<0.001	0.972 (0.969–0.976)
Low-density lipoprotein (LDL) (mg/dL)	25,371	108.6 ± 30.2	0.1	<0.001	1.024 (1.020–1.027)	0.044	<0.001	1.006 (1.004–1.008)
LDL cholesterol calculated (mg/dL)	25,324	107.3 ± 30.6	0.126	<0.001	1.025 (1.022–1.029)	0.047	<0.001	1.006 (1.004–1.008)
Triglycerides (mg/dL)	39,482	104.5 ± 64.1	0.091	<0.001	1.009 (1.008–1.011)	0.062	<0.001	1.004 (1.003–1.005)
Very low density lipoprotein (VLDL) (mg/dL)	39,378	20.6 ± 11.2	0.091	<0.001	1.060 (1.052–1.068)	0.062	<0.001	1.024 (1.020–1.028)
Non-HDL cholesterol (mg/dL)	21,410	129.9 ± 35.3	0.124	<0.001	1.026 (1.022–1.029)	0.061	<0.001	1.007 (1.005–1.008)

**Table 5:** Multivariate linear regression analysis for SDU-D (delivered procedures) as the dependent variable with statistically significant independent parameters including collinearity statistics (statistically significant parameters are in **bold**).

Parameter	B	Std. Error	*p* Value	Exp(B) and 95% Wald Confidence Interval for	Collinearity Statistics	
					Tolerance	VIF
(Intercept)	2.93	1.35	0.030			
Age (years)	0.12	13.41	**0.002**	**1.012 (1.004–1.020)**	0.605	1.653
Sex: male vs. female	0.06	0.04	0.097	1.07 (0.98–1.15)	0.959	1.042
Socio-economic status (SES)—low vs. high	2.28	0.09	**<0.001**	**9.84 (8.23–11.77)**	0.938	1.066
Socio-economic status (SES)—medium vs. high	0.66	0.03	**<0.001**	**1.95 (1.81–2.09)**	0.940	1.064
Locality of residence: urban non-Jewish vs. urban Jewish	−0.49	0.05	**<0.001**	**0.61 (0.55–0.67)**	0.978	1.022
Locality of residence: rural vs. urban non-Jewish	0.25	0.43	0.557	1.29 (0.55–3.04)	0.974	1.027
Birth country: Western Europe vs. native Israeli	0.78	0.06	**<0.001**	**2.19 (1.93–2.48)**	0.980	1.020
Birth country: Eastern Europe and FSU vs. native Israeli	1.49	0.15	**<0.001**	**4.45 (3.32–5.96)**	0.944	1.006
Birth country: Asia vs. native Israeli	0.51	0.29	0.077	1.67 (0.94–2.95)	0.998	1.002
Birth country: Ethiopia vs. native Israeli	0.70	0.13	**<0.001**	**2.03 (1.54–2.65)**	0.992	1.009
Birth country: Africa vs. native Israeli	−0.04	0.35	0.905	0.95 (0.47–1.92)	0.997	1.003
Birth country: North America vs. native Israeli	−0.78	0.12	**<0.001**	**0.45 (0.35–0.58)**	0.994	1.006
Birth Country: South America vs. native Israeli	−0.15	0.20	0.457	0.85 (0.57–1.28)	0.998	1.002
Brushing teeth less than once a day	0.75	0.05	**<0.001**	**2.11 (1.90–2.35)**	0.986	1.014
Consumption of cariogenic diet	0.12	0.04	**0.002**	**1.13 (1.05–1.23)**	0.729	1.372
Consumption of sweetened beverages	0.64	0.04	**<0.001**	**1.89 (1.75–2.05)**	0.725	1.380
Smoking	0.22	0.09	**0.015**	**1.25 (1.04–1.50)**	0.784	1.275
Hypertension	0.24	0.11	0.840	1.02 (0.81–1.28)	0.918	1.089
Diabetes mellitus	0.37	0.35	0.290	0.68 (0.34–1.38)	0.946	1.058
Impaired glucose tolerance (IGT)	0.78	0.65	0.226	2.20 (0.61–7.87)	0.974	1.027
Hyperlipidemia	0.22	0.22	0.318	1.25 (0.80–1.93)	0.962	1.040
Obesity	0.38	0.09	**<0.001**	**1.47 (1.23–1.76)**	0.706	1.417
Cardiovascular disease	0.002	0.11	0.998	1.00 (0.80–1.25)	0.939	1.065
Fatty liver	0.24	0.21	0.264	1.26 (0.83–1.93)	0.904	1.106
Transient ischemic attack (TIA)	0.83	0.64	0.192	2.31 (0.65–8.13)	0.972	1.029

**Table 6:** Multivariate linear regression analysis for SDU-P (planned procedures) as the dependent variable with statistically significant independent parameters including collinearity statistics (statistically significant parameters are in **bold**).

Parameter	B	Std. Error	*p* Value	Exp(B) and 95% Wald Confidence Interval for	Collinearity Statistics	
					Tolerance	VIF
(Intercept)	6.30	1.43	<0.001			
Age (years)	0.17	0.004	**<0.001**	**1.19 (1.18–1.20)**	0.606	1.650
Sex: male vs. female	0.06	0.04	0.116	1.07 (0.98–1.16)	0.961	1.041
Socio-economic status (SES)—low vs. high	1.32	0.09	**<0.001**	**3.76 (3.12–4.54)**	0.940	1.064
Socio-economic status (SES)—medium vs. high	0.61	0.03	**<0.001**	**1.85 (1.71–1.99)**	0.958	1.044
The locality of residence: urban non-Jewish vs. urban Jewish	−0.35	0.05	**<0.001**	**0.70 (0.63–0.78)**	0.974	1.026
The locality of residence: rural vs. urban Jewish	0.57	0.46	0.215	1.77 (0.71–4.41)	0.978	1.022
Birth country: Western Europe vs. native Israeli	0.81	0.06	**<0.001**	**2.26 (1.97–2.58)**	0.980	1.020
Birth country: Eastern Europe and FSU vs. native Israeli	1.23	0.16	**<0.001**	**3.43 (2.52–4.67)**	0.994	1.006
Birth country: Asia vs. native Israeli	−1.12	0.30	0.887	0.88 (0.48–1.62)	0.998	1.002
Birth country: Ethiopia vs. native Israeli	0.66	0.14	**<0.001**	**1.93 (1.45–2.57)**	0.992	1.008
Birth country: Africa vs. native Israeli	0.23	0.37	0.531	1.26 (0.60–2.64)	0.997	1.003
Birth country: North America vs. native Israeli	−0.64	0.13	**<0.001**	**0.52 (0.40–0.68)**	0.994	1.006
Birth Country: South America vs. native Israeli	0.22	0.21	0.318	1.24 (0.81–1.90)	0.998	1.002
Brushing teeth less than once a day	0.46	0.05	**<0.001**	**1.58 (1.42–1.77)**	0.986	1.014
Consumption of sweetened beverages	0.27	0.03	**<0.001**	**1.31 (1.22–1.41)**	0.986	1.014
Smoking	0.30	0.09	**0.002**	**1.36 (1.12–1.64)**	0.785	1.274
Hypertension	0.08	0.12	0.504	1.08 (0.85–1.38)	0.918	1.089
Diabetes mellitus	0.44	0.37	0.239	1.56 (0.74–3.27)	0.946	1.058
Impaired glucose tolerance (IGT)	2.00	0.69	**0.004**	**7.40 (1.91–28.57)**	0.974	1.027
Hyperlipidemia	0.05	0.23	0.806	1.06 (0.66–1.68)	0.962	1.040
Obesity	0.16	0.09	0.086	1.18 (0.98–1.43)	0.706	1.416
Cardiovascular disease	0.001	0.12	0.992	1.00 (0.79–1.26)	0.939	1.065
Fatty liver	0.60	0.22	**0.008**	**1.82 (1.17–2.84)**	0.904	1.106
Obstructive sleep apnea (OSA)	1.61	0.37	**<0.001**	**5.05 (2.40–10.63)**	0.974	1.027
Transient ischemic attack (TIA)	0.93	0.68	0.157	2.61 (0.69–9.90)	0.972	1.029
Stroke	0.16	0.68	0.809	1.17 (0.31–4.47)	0.971	1.030
Deep vein thrombosis (DVT)	1.72	0.66	**0.009**	**5.61 (1.53–20.83)**	0.972	1.029

## Data Availability

Data sharing not applicable.

## Abbreviations

ADA-CDT	American Dental Association Current Dental Terminology
CI	Confidence interval
CPR	Clinical patient record
DOME	Dental, Oral, Medical Epidemiological
DPR	Dental patient record
DVT	Deep vein thrombosis
IDF	Israel Defense Forces
IGT	Impaired glucose tolerance
MetS	Metabolic syndrome
OSA	Obstructive sleep apnea
OR	Odds ratio
RCT	Root canal treatment
SDU	Standard dental unit
SDU-P	Standard dental unit—performed
SDU-D	Standard dental unit—delivered
SES	Socio-economic status
SNODENT	Systematized Nomenclature of Dentistry
TIA	Transient ischemic attack
WHO	World Health Organization (WHO)
